# Lack of resolution of inflammation marks malignant pleural mesothelioma progression

**DOI:** 10.20517/cdr.2026.33

**Published:** 2026-07-23

**Authors:** Veronica Gatti, Matti van Welzen, Alessandro Napoli, Marc Dubourdeau, Marta Tiberi, Andrea Marra, Giorgia Giorgi, Clémence Gély, Veronica Ceci, Olaf Wolkenhauer, Tommaso Mazza, Shailendra Gupta, Mario Cioce, Valerio Chiurchiù, Vito Michele Fazio

**Affiliations:** ^1^Laboratory of Molecular Medicine and Biotechnology, Department of Medicine, University Campus Bio-Medico of Rome, Rome 00128, Italy.; ^2^Department of Systems Biology and Bioinformatics, University of Rostock, Rostock 18051, Germany.; ^3^Computational Biology and Bioinformatics Unit, Fondazione Policlinico Universitario A. Gemelli, IRCCS, Rome 00168, Italy.; ^4^Ambiotis SAS, Toulouse 31400, France.; ^5^Laboratory of Resolution of Neuroinflammation, LIFE Institute for Research and Health Care, IRCCS Santa Lucia Foundation, Rome 00179, Italy.; ^6^Institute of Translational Pharmacology, National Research Council of Italy (CNR), Rome 00133, Italy.; ^7^Laboratory of Oncology, IRCCS Casa Sollievo della Sofferenza, San Giovanni Rotondo 71013, Italy.; ^#^These authors share senior authorship.

**Keywords:** Specialized pro-resolving mediators, inflammation resolution, mesothelioma, chemoresistance, lipidomics

## Abstract

**Aim:** Malignant pleural mesothelioma (MPM) is a paradigmatic inflammation-associated cancer and a major therapeutic challenge due to its resistance to treatment. Inflammation is thought to promote both tumor progression and therapy resistance. Senescence-associated secretory phenotype (SASP)-mediated, chemotherapy-induced release of arachidonic acid (AA), eicosapentaenoic acid (EPA), and docosahexaenoic acid (DHA) contributes to pemetrexed resistance in MPM cells *in vitro*.

**Methods:** Pro-inflammatory mediators (PIMs) and specialized pro-resolving mediators (SPMs) were quantified in pleural exudates using liquid chromatography-tandem mass spectrometry. Single-cell RNA-sequencing datasets were analyzed to assess the distribution of SPM-producing enzymes between mesothelial and mesothelioma cells in both tumor tissue and uninvolved pleura. Least Absolute Shrinkage and Selection Operator regression (LASSO) was used to derive a composite prognostic score (CPS) from SPM-related gene expression.

**Results:** Chemotherapy-treated patients exhibited a reduction in SPMs - including resolvins, protectins, and maresins - alongside increased prostaglandin levels. Single-cell analysis revealed differential partitioning of SPM-producing enzymes, with lower expression in mesothelioma cells compared with mesothelial cells. A five-gene CPS derived from this lipid mediator landscape independently predicted overall survival beyond conventional clinical parameters.

**Conclusion:** Chemotherapy alters the lipid mediator composition of pleural exudates in MPM by reducing SPMs, thereby disrupting resolution pathways. This effect is consistent with an intrinsic imbalance in expression between mesothelial and mesothelioma cells. Together, these findings suggest that impaired resolution of inflammation contributes to a pro-tumorigenic, pro-inflammatory, and chemoresistant microenvironment in MPM, with potential prognostic implications.

## INTRODUCTION

Malignant pleural mesothelioma (MPM) is a cancer of the mesothelial lining of the lung, characterized by insidious onset, long latency, and poor survival outcomes^[1]^. Its development is strongly linked to chronic inflammation triggered by asbestos fibers and nanoparticles, which contribute to an inflammatory and immunosuppressive tumor microenvironment^[2]^. Upon asbestos fiber uptake, mesothelial cells undergo necrosis, leading to the release into the extracellular space of damage-associated molecular pattern (DAMP) components, such as the high-mobility group box 1 (HMGB1)^[3]^. Those factors induce sustained activation of proinflammatory pathways that drive cancer progression^[4]^. While asbestos remains a main etiologic factor, a significant subset - approximately 12%-20% - of mesotheliomas is driven primarily by genetic alterations, particularly involving BRCA1-associated protein 1 (BAP1), a tumor suppressor inactivated in mesothelioma^[5]^. Importantly, inflammation-driven pathways intersect with genetic susceptibility: for example, BAP1 loss potentiates the action of HMGB1, further amplifying its protumorigenic functions^[6]^, raising the possibility that complex genetic-environmental interactions sustain the disease. The World Health Organization (WHO) defines three main histologic subtypes of pleural mesothelioma: epithelioid, sarcomatoid, and biphasic, which comprises both epithelioid and sarcomatoid components. Epithelioid mesothelioma represents 60% of cases and is associated with a more favorable prognosis^[7]^. Although immunotherapy has shown promise, especially for the non-epithelioid histotype^[8]^, chemotherapy with cisplatin and pemetrexed remains the most common treatment for patients. Nevertheless, both progression-free and overall survival (OS) remain unsatisfactory^[9,10]^, with an average 5-year OS of 15%^[11]^. This is largely due to the near-universal emergence of chemoresistance^[12,13]^.

Resistance to pemetrexed and platinum in MPM arises from complex adaptive processes, including the emergence of resistant cell populations driven by stress-induced phenotypes such as the senescence-associated secretory phenotype (SASP), which involves the release of pro-inflammatory chemokines and lipids following chemotherapy exposure^[14,15]^. In more detail, we demonstrated that arachidonic acid (AA), eicosapentaenoic acid (EPA), and docosahexaenoic acid (DHA) are actively released by mesothelioma cells in a transformation- and chemotherapy-dependent manner. We also showed that AA treatment promotes chemoresistant traits by increasing the proportion of aldehyde dehydrogenase (ALDH)-positive MPM cells^[15]^.

AA metabolism is a major source of pro-inflammatory mediators (PIMs)^[16]^and is tightly interconnected with EPA and DHA metabolism, which generates specialized pro-resolving mediators (SPMs). These include resolvins, protectins, and maresins - lipid mediators that prevent the transition to chronic inflammation and promote tissue regeneration^[17,18]^. Recent murine studies suggest that SPMs may also exert anti-cancer effects, including reduced tumor proliferation and metastasis, potentially through enhanced clearance of chemotherapy-induced tumor debris^[19]^. Although the role of inflammation in cancer progression is well recognized, the contribution of impaired resolution pathways to therapy response remains poorly understood^[20]^.

Building on our previous *in vitro* observations^[15]^, we used metabolomic and lipidomic profiling to investigate the landscape of lipid mediators in pleural exudates from chemotherapy-naïve and chemotherapy-treated MPM patients, including AA-, EPA-, and DHA-derived metabolites. We confirmed that MPM is characterized by a pro-inflammatory environment and, for the first time, identified - through a geno-lipidomic approach - disruptions in the resolution phase of inflammation associated with both transformation status and chemotherapy exposure. Failure to engage these pro-resolving pathways may promote chronic inflammation, and the behavior of the inflamed MPM microenvironment under chemotherapy-induced stress has remained largely unexplored in this context.

## METHODS

### Patient selection and source of MPM specimens

MPM pleural exudates (*n* = 19) were obtained from Mesobank, a Research Ethics Committee-approved Research Tissue Bank. The MPMs were of epithelioid histology, except for one with biphasic histology. Demographic and clinical data of the patients are reported in Table 1. All patients provided written informed consent, and samples were anonymized. Mesobank is supported by Asthma and Lung UK, The Victor Dahdaleh Foundation, and the June Hancock Mesothelioma Research Fund.

**Table 1:** Basic clinical information of the MPM patients from whom pleural exudates were obtained

**Gender**	**Age at diagnosis**	**Histotype**	**Asbestos exposure**	**Treatment **	**Cycles**
M	55	Epithelioid	yes	yes	6
M	76	Epithelioid	yes	yes	6
M	75	Epithelioid	yes	yes*	6
M	65	Epithelioid	yes	yes	4
M	73	Epithelioid	yes	yes	4
M	79	Epithelioid	no	yes	2
F	71	Biphasic	no	yes	2
M	74	Epithelioid	yes	yes	2
M	75	Epithelioid	no	yes	2
M	79	Epithelioid	yes	yes	2
M	93	Epithelioid	yes	yes	2
M	69	Epithelioid	yes	no	
M	74	Epithelioid	yes	no	
M	79	Epithelioid	yes	no	
F	71	Epithelioid	yes	no	
M	75	Epithelioid	yes	no	
M	70	Epithelioid	yes	no	
M	79	Epithelioid	yes	no	
M	71	Epithelioid	yes	no	

^*^This patient received pemetrexed + carboplatin (instead of pemetrexed + cisplatin). MPM: Malignant pleural mesothelioma.

### Targeted-metabololipidomics analysis of lipid mediators

Lipid mediators were quantified in the pleural fluid of patients with or without Pemetrexed chemotherapy. All samples were fast frozen and stored at -80 °C for a maximum of 6 months before lipidomic analysis to prevent alteration of lipid content. Fifty-six oxylipins and metabolites of AA, EPA, and DHA were quantified by liquid chromatography-tandem mass spectrometry (LC-MS/MS) [Supplementary Table 1]. The extraction and analysis were performed as previously described^[21]^. Briefly, lipid mediators were extracted from samples by solid-phase extraction (SPE) and eluted with a mixture of methyl formate and methanol (MeOH). After solvent evaporation, samples were dissolved in MeOH:H_2_O, 50:50 (v/v), and injected into an ultra-high-performance liquid chromatography (U-HPLC) Exion LCAD system coupled to a 6500+ QTRAP (AB SCIEX GmbH, Zug, Switzerland) mass spectrometer. The lipids were separated on a Kinetex C18 column (100 mm × 2.1 mm, 1.7 µm, Phenomenex) and detected by negative electrospray ionization in multiple reaction monitoring (MRM) mode. When authentic standards were available, the identification of the lipid mediators in the samples was based on their retention times with those of the pure standards injected under the same conditions. Calibration curves were obtained using the same authentic standards, and quantification was carried out based on peak areas from MRM transitions using a linear model weighted by 1/X (X, the nominal concentration). For 21-HDOHE, 4(S),14(S)-DiHDOHE, RvD6, 18(S)-RvE3, 18(R)-RvE3 identification was based on theoretical MRM transitions established on the basis of the molecule’s structure, and quantification was carried out using a pure standard with a similar structure. Peak detection, integration, and quantitative analysis were performed using Sciex OS MQ software. Quantification results were expressed in pg/mL and detailed in Supplementary Table 2.

### Data preprocessing and survival analysis in TCGA mesothelioma dataset

The Mesothelioma gene expression datasets used in this study were downloaded from The Cancer Genome Atlas (TCGA) (https://www.cancer.gov/tcga), specifically from the GDC TCGA Mesothelioma (MESO) project. Gene expression data were provided as FPKM values. Survival information was defined as the OS of patients, i.e., the time from diagnosis to death, regardless of cause. **Deconvolution and Statistical Analysis in TCGA Mesothelioma Dataset.** The gene expression data were used to perform bulk RNA-seq deconvolution using CIBERSORTx, a tool for cell-type profiling. CIBERSORTx was employed with the LM22 gene signature, which is a predefined gene signature matrix for 22 immune cell types, to estimate the relative proportions of different immune cell populations in the bulk tumor samples. Following deconvolution, the patients were stratified into “low-expressing” and “high-expressing” groups, based on the composite signature calculated in the previous steps. To compare immune cell-type differences between the two groups, a Mann-Whitney *U* test was performed using Python’s scipy stats module. Boxplots were drawn for immune cell types whenever the Mann-Whitney U test revealed statistically significant differences (*P* < 0.05).

### Differential gene expression analysis and pathway enrichment

To further investigate the transcriptional differences between the highly/lowly Composite Signature-expressing groups, a differential gene expression analysis was performed on the FPKM-UP expression data with the LIMMA package. To identify differentially expressed genes (DEGs), the following hard filters were applied: |log2FC| > 0.5 and adjusted *P*-value < 0.05. *P*-values were adjusted using the Benjamini-Hochberg (BH) method. IPA QIAGEN Inc.(https://www.qiagenbioinformatics.com/products/ingenuity-pathway-analysis) and KEGG pathway enrichment analysis were performed to explore the biological implications of the resulting DEGs.

### Feature selection and composite signature development

From the GDC-TCGA mesothelioma dataset described above, a panel of genes associated with lipid metabolism and oxidative stress pathways was selected for analysis: *PTGR1*, *ALOX12*, *LTA4H*, *EPHX2*, *EPHX1*, *CYP3A4*, *ALOX15*, *GPX4*, *GSTM4*, and *CYP4F11*. Categorical variables, such as “treatment status” and “diagnosis”, were converted into binary variables through one-hot encoding. A binary variable was created to define the survival outcome based on the median OS time, classifying patients with survival times above or below the median. The dataset was stratified into two groups based on treatment status: patients receiving and not receiving therapy. Lasso regression with 10-fold cross-validation was applied separately to the treated and untreated groups to identify genes significantly associated with the binary survival outcome. Lasso regression was implemented using the sklearn Python library v1.2.2. Genes with non-zero Lasso coefficients were considered predictive of survival. The intersection of genes selected from the treated and untreated groups was computed to identify shared prognostic features. A bootstrap analysis with 100 resampling iterations was performed to evaluate the selected genes’ robustness. For each iteration, the dataset was resampled, and the Lasso model was refitted. The coefficients of the genes identified in both groups were recorded across iterations. The mean of the coefficients was calculated to assess their stability and significance. A composite prognostic score (CPS) was derived as a linear combination of the expression values of the overlapping genes, weighted by their mean Lasso coefficients obtained from the bootstrap analysis. The CPS was used to stratify patients into high- and low- risk groups based on the median value of the signature.

### Survival analysis and prognostic modeling

KM-plotter (http://kmplot.com/analysis/) was employed to assess the impact of mRNA levels of representative SPM-producing enzymes in MPM patients from the GDC-TGCA mesothelioma database. Additionally, Kaplan-Meier survival analysis was performed to compare survival outcomes between high-risk and low-risk groups. The Log-Rank test was used to evaluate the statistical significance of differences in survival curves between the two groups. The Kaplan-Meier survival curves were visualized to illustrate the separation between risk groups. To further confirm the prognostic significance of the composite signature, a Cox proportional hazards model was fitted with survival time, event status, and the composite signature as predictors. Kaplan-Meier curves, Log-Rank tests, and Cox regression were conducted using the lifelines Python library v0.27.8. To evaluate the predictive performance of the CPS, we performed time-dependent receiver operating characteristic (ROC) analysis at 12, 24, and 36 months of overall survival. Risk scores were derived from the multivariate Cox model, with higher partial hazard scores indicating increased risk. The 24-month timepoint was chosen as the primary threshold for further diagnostic assessment, as it ensured sufficient observed deaths (*n* = 50) and patients still at risk (*n* = 31). By comparison, the 12-month analysis had fewer events, and at 36 months, only 14 patients, limiting robustness for calibration and sensitivity/specificity estimation. To further characterize the model’s performance at 24 months, sensitivity and specificity were computed using Youden’s index to determine the optimal threshold, and a calibration plot was generated.

### Statistics

Results are expressed as means ± SEM and compared using a nonparametric Mann-Whitney two-sided test in GraphPad Prism 9 (GraphPad Software, San Diego, CA). Given the exploratory nature of the lipidomic analysis, which involved 56 lipid species and multiple pairwise comparisons among the three experimental groups (Naive, Chemo 2 cycles, and Chemo > 4 cycles), *P* values obtained from Mann-Whitney *U* tests were corrected for multiple testing using the Benjamini-Hochberg false discovery rate (FDR) method. The adjustment was performed in R software (version 4.6.0). Lipids with an adjusted *P*-value (FDR) < 0.05 were considered statistically significant. Principal component analysis (PCA) was created using the freely available R software. For multiple correlation analysis, the free StatCalculator was used (https://www.statscalculators.com/), with control for multicollinearity. For the OS analysis, Kaplan-Meier survival curves were generated and compared between untreated and chemotherapy-treated patients using the log-rank test, implemented in the lifelines Python library (v0.27.8). For multiple correlation analysis, the free StatCalculator was used (https://www.statscalculators.com/), with control for multicollinearity.

Single-cell RNA sequencing (scRNA-seq) data processing and *in silico* integration into Inflammation-resolution networks. Single-cell RNA-seq profiles (GSE243446 and GSE190597) of human pleura with preprocessed and library-size-normalized read counts (*q*) were downloaded from published studies^[22]^. Based on canonical markers and signature genes cross-referenced to known markers, the dataset includes many different mesothelial, immune, and stromal cells. To ensure the analysis aligns with the data generated in the present study, patients 1 and 3 from the GSE243446 dataset were excluded due to comorbidities. The dataset GSE190597 includes 13 samples from three histological types of tumors: sarcomatoid, biphasic, and epithelioid, and encompasses 15 distinct cell types, including both malignant and mesothelial cells. Log1p normalization, total-count normalization, and scaling of the data were performed using the scanpy-python package. We applied a Gaussian-Mixed-Model (GMM) before scaling to divide the samples into expressing and unexpressed cells for each gene, identifying a suitable read-count threshold that defines a gene as expressed or not. We downloaded manually curated networks from the “Biosynthesis of PIM and SPM from AA”, “Biosynthesis of PIM and SPM from DHA”, and “Biosynthesis of PIM and SPM from EPA” pathways of our previously published “Atlas of Inflammation Resolution” (AIR) platform^[23]^. We manually mapped the lipids analyzed in the present study to molecules in the network to avoid naming-convention mismatches. For each of the lipids and, additionally, for each lipid class that is represented as phenotype elements in the network, we identified all sets of genes corresponding to enzymes that could catalyze their synthesis from either one of the substrates AA, DHA, or EPA as described in previous works^[24]^. Finally, we considered a lipid mediator to be synthesizable in a given cell if the cell expresses all genes in at least one of the gene sets. To identify cell clusters of similar lipid mediator (LM) synthesis, we first reduced the data to only genes of enzymes occurring in the lipid mediator network and the samples to only cells where at least one lipid mediator was identified as synthesizable. We then applied Uniform Manifold Approximation and Projection (UMAP) dimensionality reduction from the scanpy Python package on the reduced dataset. Clusters in the UMAP embedding were identified using Leiden graph-clustering with a manually selected resolution parameter of 0.275. A dot plot was generated using the DotPlot function from the Seurat package to visualize the expression of selected genes across the mesothelial and malignant cells.

## RESULTS

### Targeted metabololipidomics of pleural fluid from MPM patients

**Figure 1:**
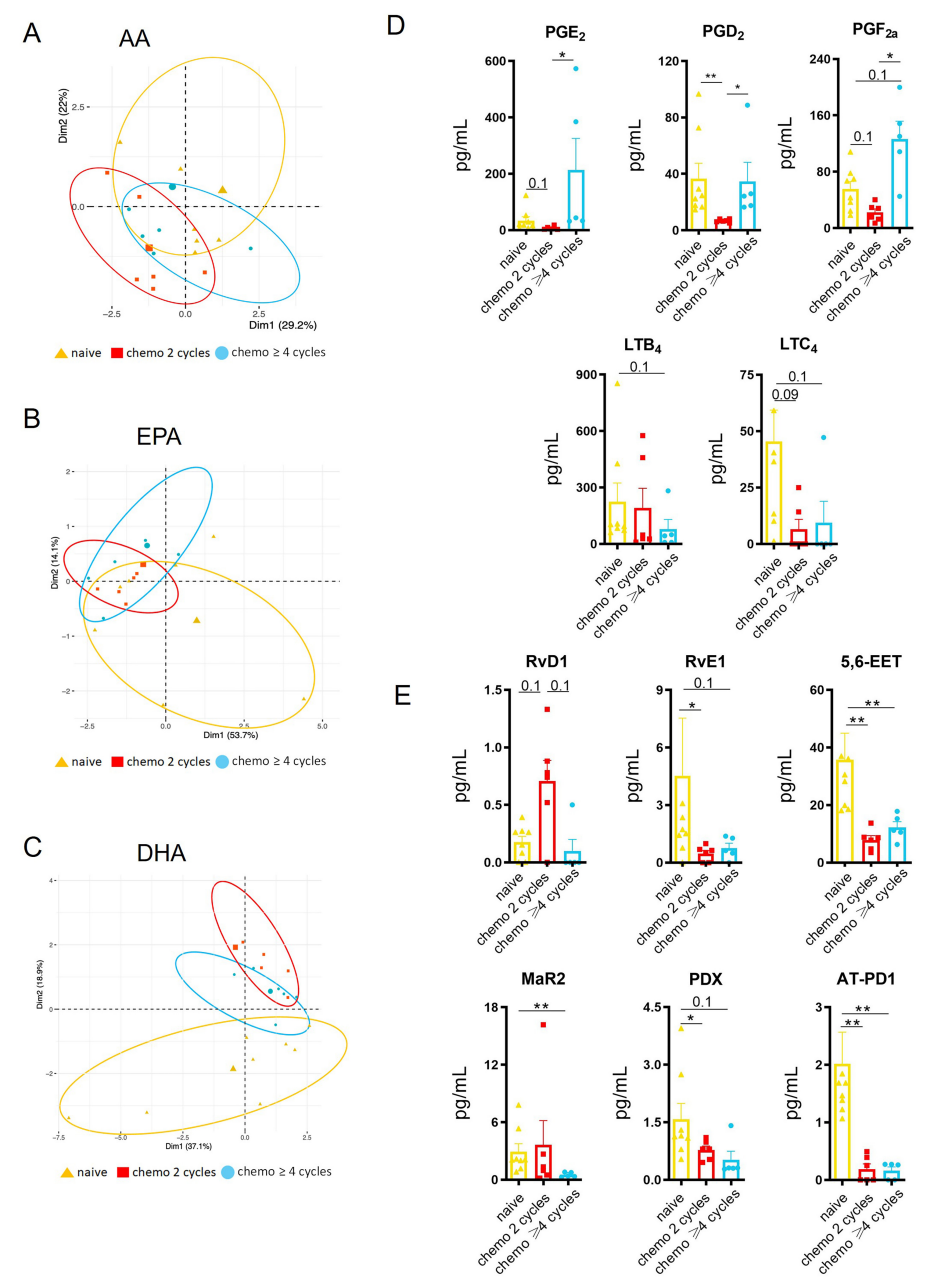
Lipid mediators are altered in pleural exudates of MPM patients treated with chemotherapy. (A-C) PCA: clustering of identified AA-, EPA-, and DHA- derived lipid mediators; (D and E) Histograms show the quantification of selected lipid mediators. Data are presented as means ± SEM (pg/mL). ^*^P < 0.05, ^**^P < 0.01. No asterisk = not significant. Please note that where indicated, *P*-values ≤ 0.1 were reported. The *P* values obtained from Anova test were adjusted for multiple comparisons using the Benjamini-Hochberg FDR correction. AA: Arachidonic acid; EPA: eicosapentaenoic acid; DHA: docosahexaenoic acid; FDR: false discovery rate.

We performed targeted metabololipidomics by liquid chromatography-tandem mass spectrometry (LC-MS/MS) on pleural fluid samples collected from 19 patients with MPM, including untreated individuals (*n* = 8) and patients who had received two to six cycles of chemotherapy (*n* = 11; cisplatin + pemetrexed, except for one patient treated with carboplatin) [Table 1 and Supplementary Figure 1]. All but one patient exhibited epithelioid histology [Table 1]. All patients in the naïve group had anamnestically reported asbestos exposure, while three of eleven patients in the treated group were not recorded as having asbestos exposure. In total, we quantified 56 oxylipins, comprising 22 PIMs derived from AA and 34 SPMs derived from AA, EPA, or DHA [Supplementary Table 1, Figure 1 and Supplementary Figure 2 for examples of MS spectra].

### Chemotherapy differentially modulates pro-inflammatory eicosanoids

Principal component analysis (PCA) revealed clustering of chemotherapy-treated patients by lipid class and precursor fatty acids (AA, EPA, DHA) [Figure 1A-C]. We next compared oxylipin levels across three patient groups (naïve, 2 cycles, ≥ 4 cycles) to identify chemotherapy cycle-dependent differences [Figure 1 and Supplementary Table 1]. Among significantly modulated PIMs (*P* < 0.05), prostaglandins PGD_2_, PGE_2_, and PGF_2_α were markedly increased in patients who had received ≥ 4 cycles [Figure 1D]. Conversely, patients treated with two cycles exhibited significantly lower levels of these prostaglandins compared with naïve individuals. Leukotrienes (LXB_4_, LTC_4_) and hydroxy-/epoxyeicosanoids (5-HETE, 5,6-EET) decreased in both chemotherapy-treated groups. Overall, chemotherapy induced a net increase in PIMs, with prostaglandins showing a clear cycle-dependent pattern. Longer treatment duration was associated with increased prostaglandins and decreased leukotrienes [Figure 1D].

### Chemotherapy differentially modulates SPMs

We next examined EPA- and DHA-derived SPMs. Among the resolvins, RvD1 increased after two cycles but decreased in the ≥ 4-cycle group [Figure 1E]. The E-resolvin RvE1, the protectins (PDx, AT-PD1), and the AA-derived 5,6-EET decreased in both treated groups [Figure 1E]. Other SPMs, such as LXB4 and RvE3, exhibited a similar pattern without reaching significance [Supplementary Table 1]. Maresin2 (MaR2) was significantly reduced only in the high-cycle group [Figure 1E]. The SPM precursors showed no statistically significant changes, although EPA-derived 18-HEPE trended downward in both treated groups, and DHA-derived 17-HDOHE and 14-HDOHE trended upward in the high-cycle group [Supplementary Table 1]. Collectively, longer-term chemotherapy reduced RvD1, RvE1, Mar2, and the protectins PDx and AT-PD1, without significantly altering precursor levels.

### SPM levels correlate with OS in MPM

**Figure 2:**
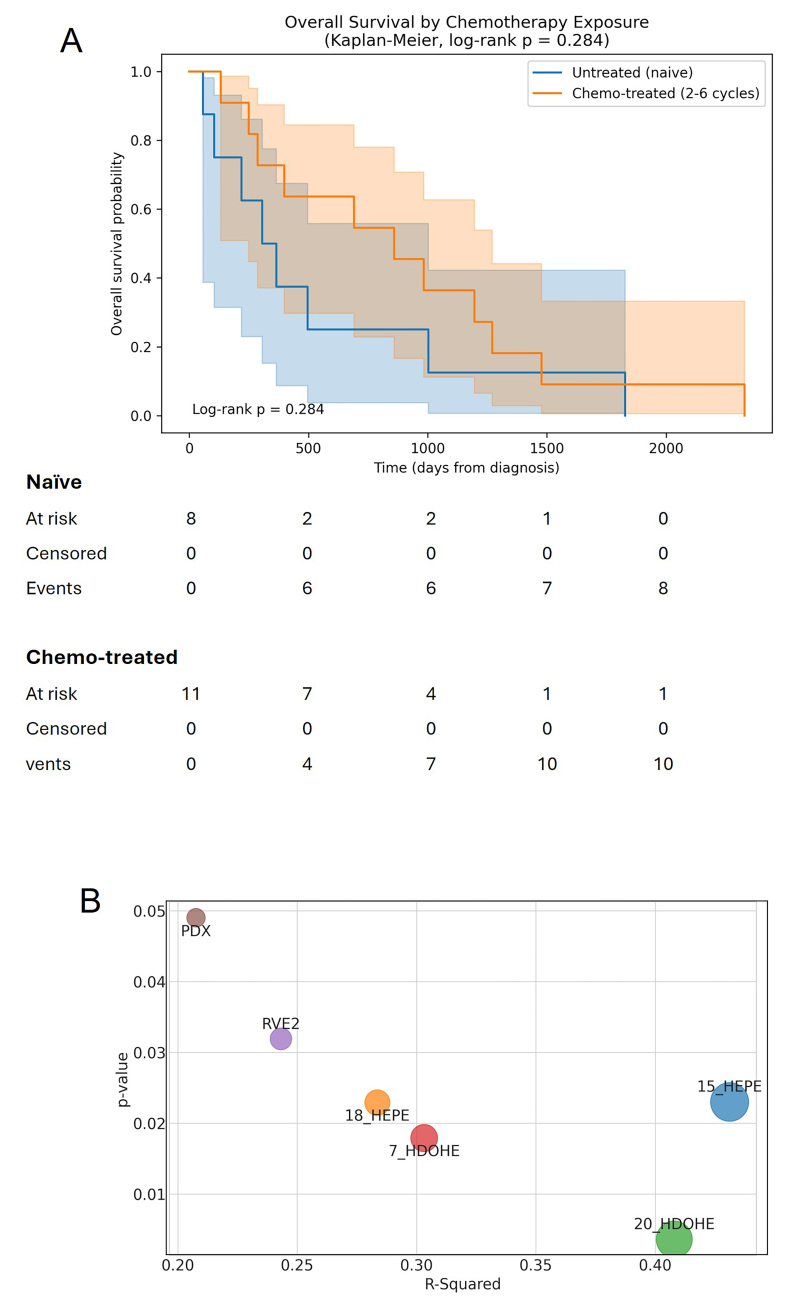
Defective SPM release in chemotherapy-treated patients does not confer a survival advantage. (A) OS of patients in this study by treatment. Graphs reporting the OS of the patients whose pleural effusions underwent lipidomic analysis. Kaplan-Meier survival curves were compared using the log-rank test (*P* = 0.284; ns: not significant); (B) Multiple correlation analysis identified the levels of the indicated oxylipins as significantly correlated with the OS of the patients. Bubble plot: X-axis: R-Squared; Y-axis: *P*-value; Bubble size: proportional to R-Squared. For the analysis, the free StatCalculator was used (https://www.statscalculators.com/).

To determine whether altered SPM profiles reflect disease aggressiveness, we evaluated OS in the same patient cohort. Chemotherapy did not improve OS, indicating that treatment-associated SPM reductions did not translate into survival differences (Kaplan-Meier analysis, log-rank test: *P* = 0.284; Figure 2A). This remained true when low- and high-cycles groups were analyzed separately [Supplementary Figure 3]. To deepen these observations, we performed multiple linear correlation analyses to evaluate whether any of the identified metabolites, including those trending (0.1 < *P* < 0.05), correlated with OS across 19 patients (both naïve and treated). We found that none of the PIMs considered exhibited a statistically significant correlation with patients’ OS. By contrast, we found that the levels of four SPM precursors (15-HEPE, 18-HEPE, 20-HDOHE, 7-HDOHE) and RvE2 and Protectin DX levels were significantly correlated with time to death [Figure 2B]. These findings suggest that impaired SPM biosynthesis may characterize MPM patients with more aggressive disease, independent of treatment.

### SPM-producing enzymes are differentially expressed in mesothelial and mesothelioma cells

**Figure 3:**
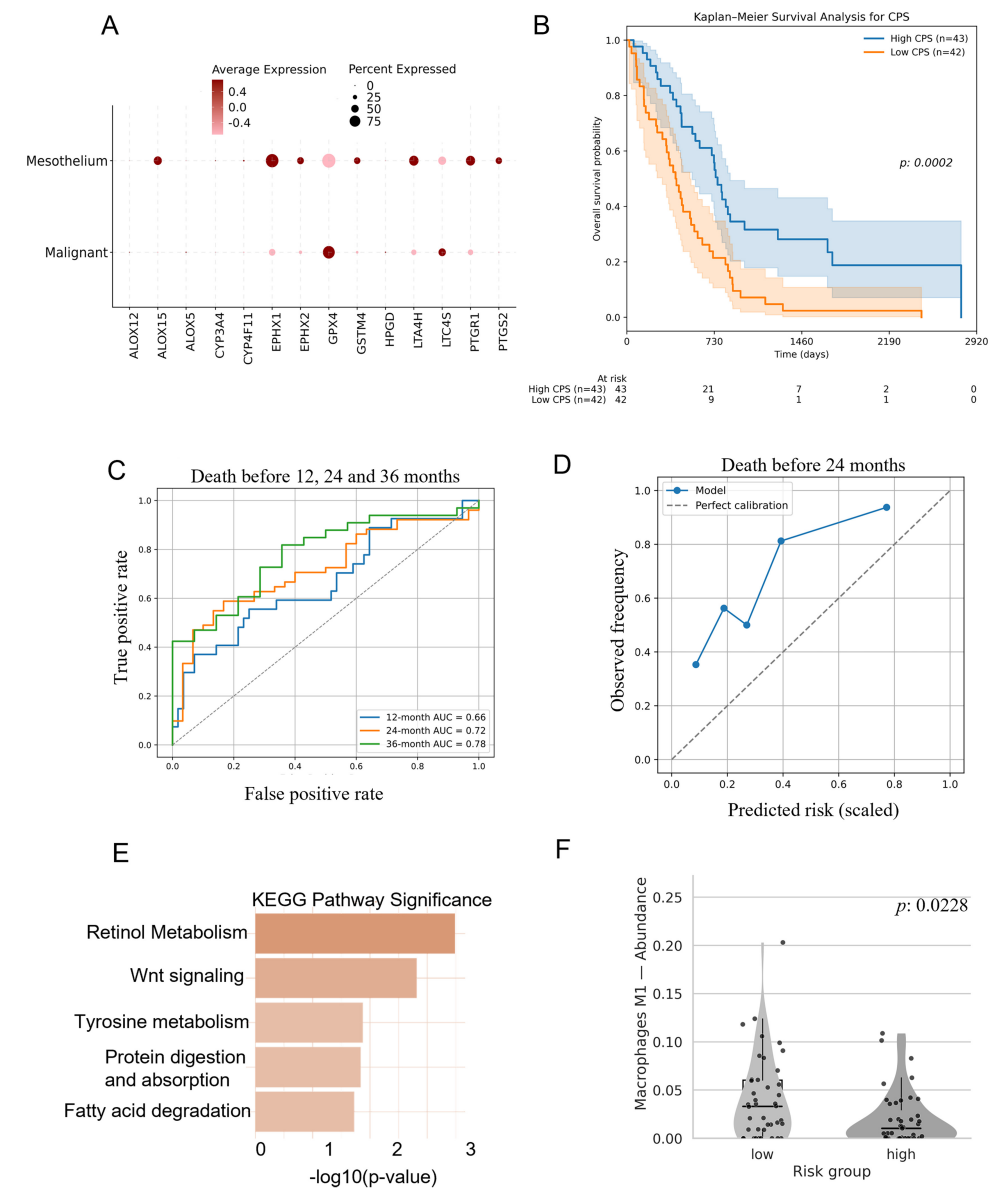
SPM-producing enzymes are downregulated in malignant mesothelial cells and predict prognosis. (A) Expression of SPM-producing enzymes in mesothelioma and intra-tumoral diploid mesothelial cells from an scRNA-seq dataset (GSE190597). Dot size indicates the percentage of cells expressing each gene, whereas color intensity indicates average normalized expression; (B) Survival probability over time of MPM patients based on the CPS. The CPS was derived by LASSO regression with bootstrap resampling and survival was compared by Kaplan-Meier analysis with the log-rank test; (C) Time-dependent ROC analysis evaluating the predictive performance of CPS risk scores for death before 12, 24, and 36 months. AUC values were 0.66 at 12 months, 0.72 at 24 months, and 0.78 at 36 months, with corresponding 95% confidence intervals of 0.53-0.79, 0.61-0.83, and 0.64-0.90, respectively, estimated by non-parametric bootstrap resampling; (D) Calibration curve of MPM patients at 24 months based on the CPS. The optimal threshold was defined using Youden’s index; (E) KEGG pathway enrichment analysis of differentially expressed genes between the high- and low-CPS groups. Differentially expressed genes were identified using limma, with *P*-values adjusted using the Benjamini-Hochberg false discovery rate correction. The more intense color of the first bar indicates higher significance; (F) Violin plots showing the distribution of M1 macrophages between the high- and low-CPS expressing groups, based on RNA-seq deconvolution results using CIBERSORTx (*P* < 0.05). Immune-cell proportions were estimated by CIBERSORTx and compared between groups by the two-sided Mann-Whitney *U* test. CPS: Composite prognostic score; SPM: specialized pro-resolving mediator; MPM: malignant pleural mesothelioma; AUC: area under the curve.

We analyzed mRNA expression of key SPM-biosynthetic enzymes using a scRNA-seq dataset containing intratumoral mesothelial diploid cells and mesothelioma cells from epithelioid MPM patients^[25]^. Mesothelioma cells exhibited reduced expression-both in magnitude and proportion of expressing cells-of several SPM-producing enzymes compared with mesothelial cells [Figure 3A]. These findings were corroborated using the AIR platform, which is based on scRNA-seq datasets from pleural tissues of non-MPM subjects and MPM patients, and revealed disrupted cellular organization in MPM tissues [Supplementary Figures 4 and 5].

### Development of an MPM composite prognostic signature

We developed a five-gene signature comprising *CYP3A4*, *ALOX15*, *EPHX1*, *GSTM4*, and *GPX4*, which stratified TCGA MPM patients into two groups. A CPS was derived as a linear combination of the expression values of the overlapping genes, weighted by their mean Lasso coefficients obtained from the bootstrap analysis. The CPS was used to stratify patients into high-risk and low- risk groups based on the median value of the signature. Those with “high” CPS exhibited better survival rates than those with “low” CPS (*P* = 0.0002) [Figure 3B]. To detail the prognostic utility of CPS, we fitted a Cox regression model, scoring treatment status, histological subtype, and pathological stage as covariates. Patients with higher CPS values had a 41% reduced risk of mortality compared with those with lower CPS values (univariate HR = 0.59; *P* < 0.005; 95%CI: 0.43-0.81). This association remained significant in the multivariate Cox model, supporting CPS as an independent predictor of OS (adjusted HR = 0.61; *P* = 0.002; 95%CI: 0.45-0.83). The time-dependent predictive performance of the CPS, as determined by receiver operating characteristic (ROC) analysis at 12, 24, and 36 months, yielded area under the curve (AUC) values of 0.66, 0.72, and 0.78, respectively, with corresponding 95% confidence intervals of 0.53-0.79, 0.61-0.83, and 0.64-0.90 [Figure 3C]. At 24 months, sensitivity was 0.588 and specificity was 0.833 [Figure 3D]. These results indicate that reduced SPM production or diminished expression of SPM-biosynthetic enzymes identifies prognostically distinct MPM patient subsets [Figure 2A].

### Pathway enrichment in Low-CPS, high-risk patients

Differential expression analysis identified 73 downregulated and 29 upregulated genes between CPS-defined groups [Supplementary Table 2]. Pathway enrichment analysis [Supplementary Tables 3 and 4] revealed significant enrichment of the retinol metabolism pathway and a trend toward enrichment of the Wnt signaling pathway (*P* = 0.07) in low-CPS “high risk” MPM patients [Figure 3E].

Immune deconvolution showed a higher proportion of M1 macrophages in the low-CPS group (*P* = 0.023) and a higher representation of plasma cells in the high-CPS group (*P* = 0.026) [Figure 3F; Supplementary Tables 5 and 6]. These findings further support the distinct biological identity of the two CPS-defined patient groups and highlight the pro-inflammatory, dysregulated microenvironment associated with low CPS and shorter OS.

## DISCUSSION

Recent murine studies indicate that SPMs exert anti-cancer effects, including reduced tumor proliferation and metastasis, potentially through suppression of tumor-associated macrophages and enhanced clearance of chemotherapy-induced tumor debris^[20]^. Here, we provide evidence that defective pro-resolving mechanisms progressively support the establishment and persistence of a chronic inflammatory milieu in MPM.

Building on our previous work demonstrating AA release from *in vitro* immortalized and pemetrexed-treated MPM cell lines, we now characterize EPA- and DHA-derived SPMs, in addition to AA-derived PIMs, in MPM patients. Our findings reveal that chemotherapy disrupts the resolution phase of inflammation, potentially contributing to therapeutic resistance and relapse, as suggested by the increased risk of death observed in patients with low CPS scores [Figure 3B].

Single-cell RNA sequencing analysis further shows that mesothelioma cells exhibit markedly reduced mRNA levels of SPM-producing enzymes. In contrast, mesothelial cells - whether diploid intra-tumoral cells or cells from normal pleura - display higher expression of these enzymes. These observations suggest an early, intrinsic defect in SPM biosynthesis within transformed mesothelioma cells, which is further exacerbated by chemotherapy. Furthermore, we have previously demonstrated that chemotherapy-dependent AA release was mediated by PLAG2^[15]^. Consistent with this, we observed higher PLAG2A mRNA levels in low-CPS, higher-risk patients identified by the SPM-related gene signature [Supplementary Table 5]. We also detected enrichment of Wnt and ALDH pathways in this cohort, in line with their established roles in MPM progression^[26-28]^. Collectively, these findings align with our earlier observations, suggesting that MPMs exhibiting reduced steady-state SPM production derived no benefit from chemotherapy [Figure 2]. These preliminary findings may have translational potential toward MPM patient stratification.

Our *in silico* analysis using the AIR platform, which also provides the added advantage of evaluating normal pleural composition, predicted an increased representation of SPM-producing macrophages in mesothelioma specimens [Supplementary Figures 4 and 5]. This may account for the residual SPM production detected in untreated MPM tissues. Further investigation is required to determine how chemotherapy may attenuate the non-cell-autonomous production of SPMs by infiltrating macrophages.

We also acknowledge several limitations of this work. First, our limited cohort of specimens does not allow us to establish a causal relationship between the observed lipidomic features and therapeutic resistance. Specifically, the comparison of OS between untreated and chemotherapy-treated patients (*n *= 8 *vs.* *n *= 11) is underpowered, and the lack of statistical significance (*P* = 0.284) should be interpreted with caution. Further, several PIMs and SPMs were not significantly modulated but showed a trend, and it is likely that in a larger cohort of specimens, their modulation would have reached statistical significance. Second, this proof-of-concept work requires mechanistic validation to verify and scrutinize the observed correlations. Thus, we acknowledge that our conclusions remain consistent with the observational nature of the data. Clarifying the causal relationships underlying the link between defective resolution and MPM resistance to therapy will require further experimental validation. In summary, our results support a reevaluation of cancer-associated inflammation through the lens of failed resolution, opening avenues for resolution-targeted therapeutic strategies to mitigate resistance to cancer therapy. Reduced expression of SPM-producing enzymes may indicate accelerated disease progression and chemotherapy ineffectiveness, potentially aiding patient stratification. In this context, Harold Dvorak’s characterization of cancers as “wounds that do not heal”^[29]^ appears prescient.

## DECLARATIONS

### Availability of data and materials

All data generated or analyzed during this study, if not included in this article and its Supplementary Materials, are available from the corresponding authors on reasonable request.

### AI and AI-assisted tools statement

During the preparation of this manuscript, the AI tool ChatGPT (version 5.5, released 2026-07-08) was used solely to cosmetically improve the graphical abstract. The tool did not influence the study design, data collection, analysis, interpretation, or the scientific content of the work. All authors take full responsibility for the accuracy, integrity, and final content of the manuscript*.*

### Copyright

© The Author(s) 2026.
