# Supplementary material for: Lack of resolution of inflammation marks malignant pleural mesothelioma progression
Source: Cancer Drug Resist. 2026 Jul 23;9:24. doi: 10.20517/cdr.2026.33 (PMC13459112; doi:10.20517/cdr.2026.33)
Supplement: Supplementary file 1 [file cdr-9-24-SupplementaryMaterials.zip › cdr9033-Supplementary Figures 1-5.pdf]

Supplementary Materials

Lack of resolution of inflammation marks malignant pleural mesothelioma progression

Veronica Gatti[1], Matti van Welzen[2], Alessandro Napoli[3], Marc Dubourdeau[4], Marta Tiberi[5], Andrea Marra[1,6], Giorgia Giorgi[5], Clémence Gély[4], Veronica Ceci[5], Olaf Wolkenhauer[2], Tommaso Mazza[3], Shailendra Gupta[2], Mario Cioce[1,6,#], Valerio Chiurchiù[5,6,#], Vito Michele Fazio[1,6,7,#]

[1]Laboratory of Molecular Medicine and Biotechnology, Department of Medicine, University Campus Bio-Medico of Rome, Rome 00128, Italy.

[2]Department of Systems Biology and Bioinformatics, University of Rostock, Rostock 18051, Germany.

[3]Computational Biology and Bioinformatics Unit, Fondazione Policlinico Universitario A. Gemelli, IRCCS, Rome 00168, Italy.

[4]Ambiotis SAS, Toulouse 31400, France.

[5]Laboratory of Resolution of Neuroinflammation, LIFE Institute for Research and Health Care, IRCCS Santa Lucia Foundation, Rome 00179, Italy.

[6]Institute of Translational Pharmacology, National Research Council of Italy (CNR), Rome 00133, Italy.

[7]Laboratory of Oncology, IRCCS Casa Sollievo della Sofferenza, San Giovanni Rotondo 71013, Italy.

[#]These authors share senior authorship.

Correspondence to: Prof. Mario Cioce, Prof. Vito Michele Fazio, Laboratory of Molecular Medicine and Biotechnology, Department of Medicine, University Campus Bio-Medico of Rome, Rome 00128, Italy. E-mail: m.cioce@unicampus.it; fazio@unicampus.it; Dr. Valerio Chiurchiù, Institute of Translational Pharmacology, National Research Council of Italy (CNR), Rome 00133, Italy. E-mail: valerio.chiurchiu@ift.cnr.it

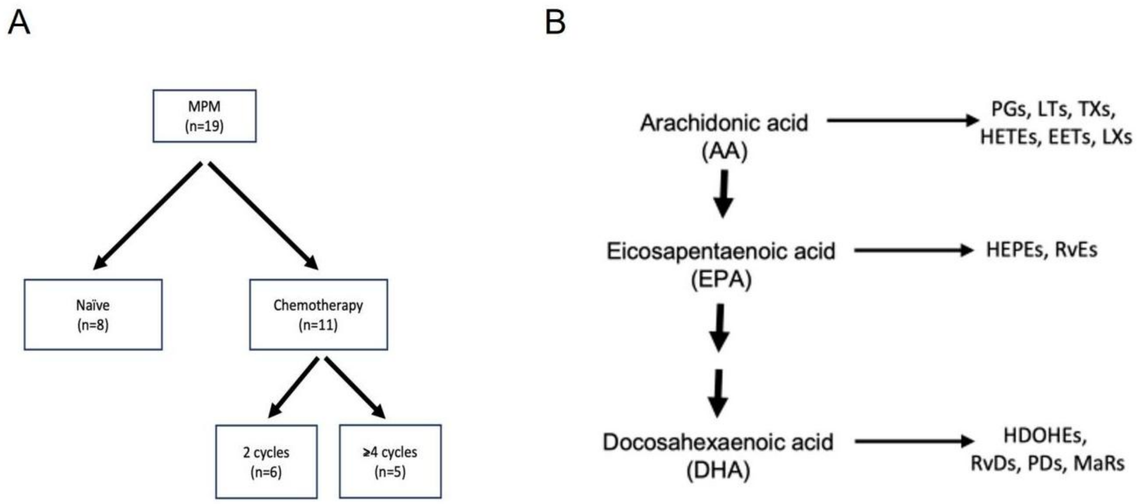

Supplementary Figure 1. Experimental scheme. (A) composition and workflow of experimental groups. MPM pleural exudates (n = 19) were obtained from patients treated (n = 6 with two cycles; n = 5 with four or more cycles) or not (n = 8) with chemotherapy. (B) Schematic representation of the classes of oxylipins and metabolites derived from AA, EPA, and DHA and analyzed in this study.

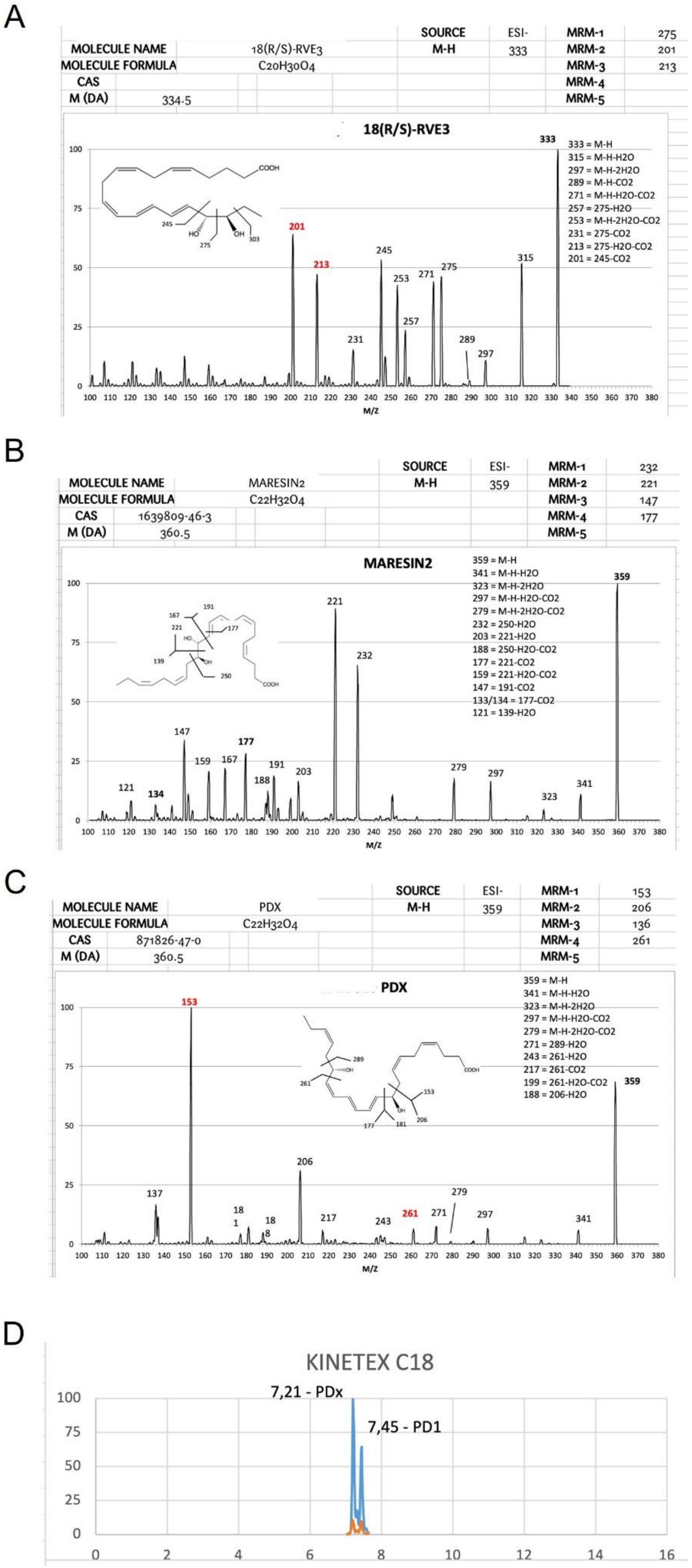

Supplementary Figure 2. Identification of SPMs. (A-C) Representative mass spectra of 18R/S-RvE3 (A), MaR2 (B), and PDX (C); (D) Distinction between PDX and PD1 isomers and their relative Multiple Reaction Monitoring (MRM).

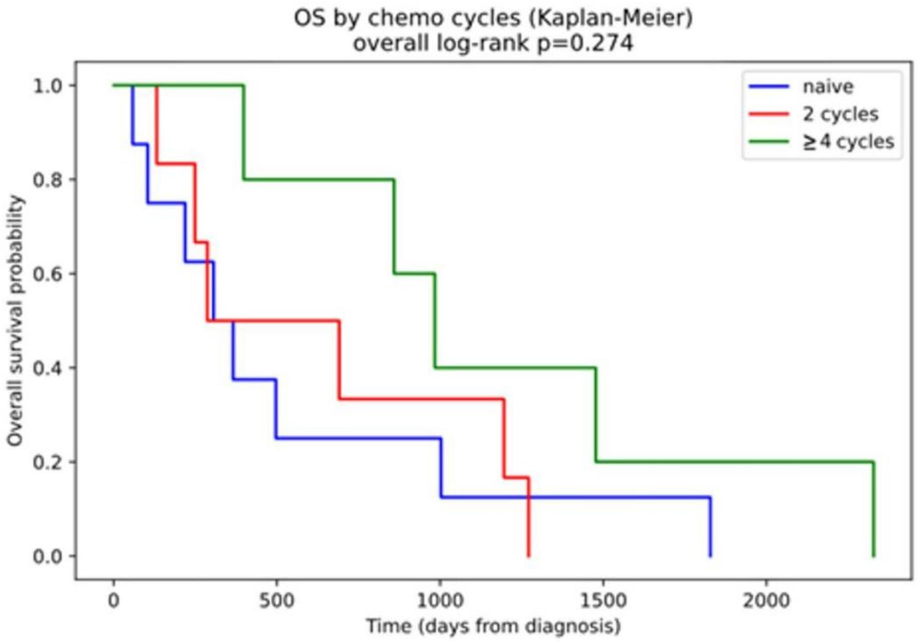

Supplementary Figure 3. Overall survival of patients enrolled in this study by treatment (number of cycles). No statistically significant differences were recorded between the three groups of MPM patients. Multiparametric log-rank test. Table: results (*P* values) of the log rank test with intergroup comparisons.

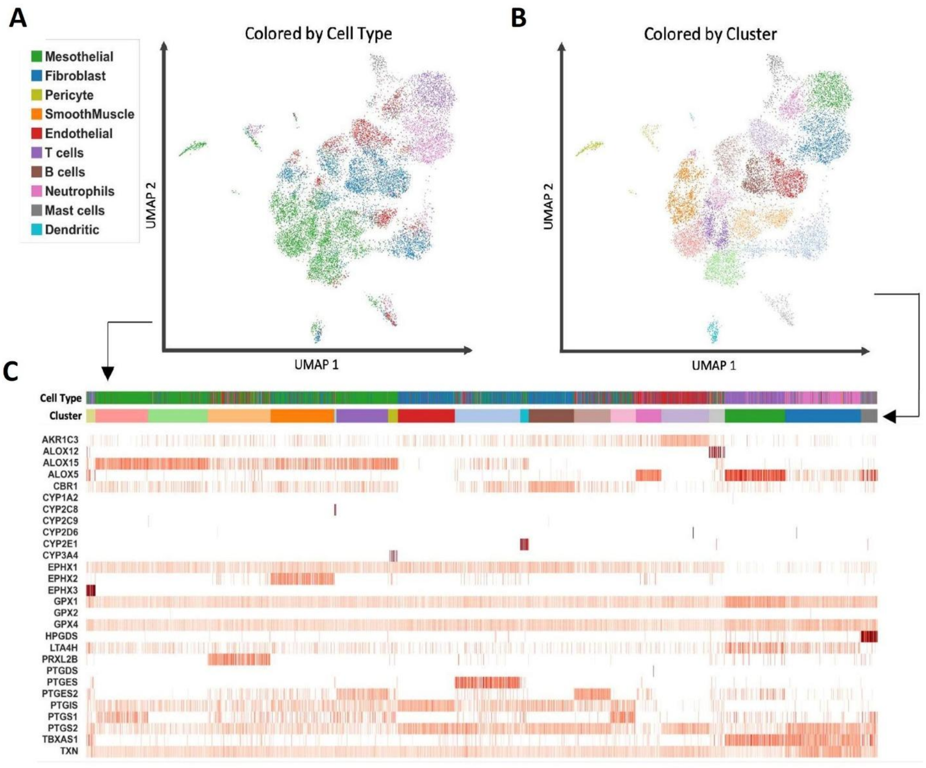

D

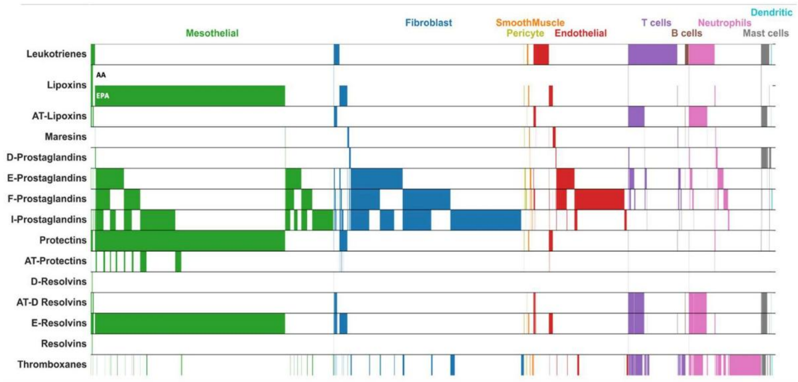

E

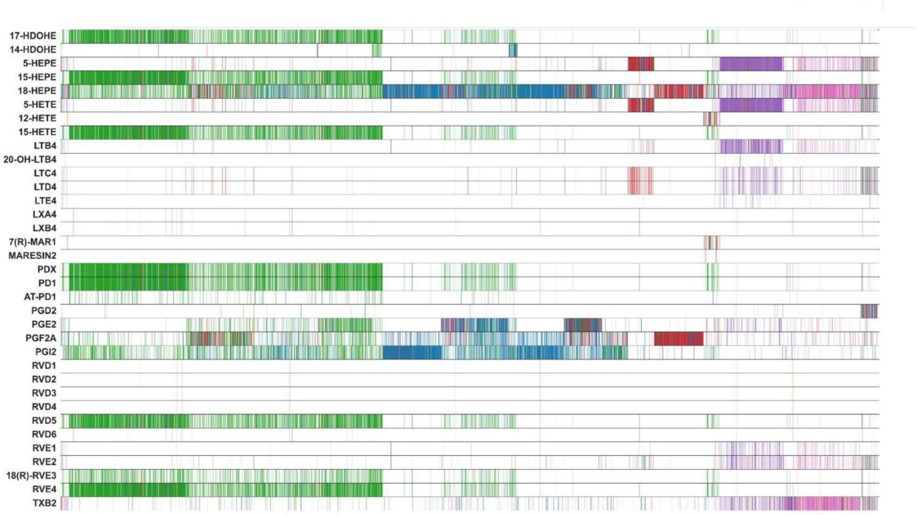

Supplementary Figure 4. Inflammation-resolution biosynthetic pathways of lipid mediators are differentially altered in MPM cell types. Predictive model. (A and B) UMAP plots are sorted by cell type and clusters in the normal pleura of control subjects; (C) Heatmaps highlighting SPM enzymes within the cell clusters of control subjects; (D and E) Heatmaps highlighting individual lipids in cells that express all required enzymes for their synthesis, as measured by targeted lipidomics in the human pleura of control subjects.

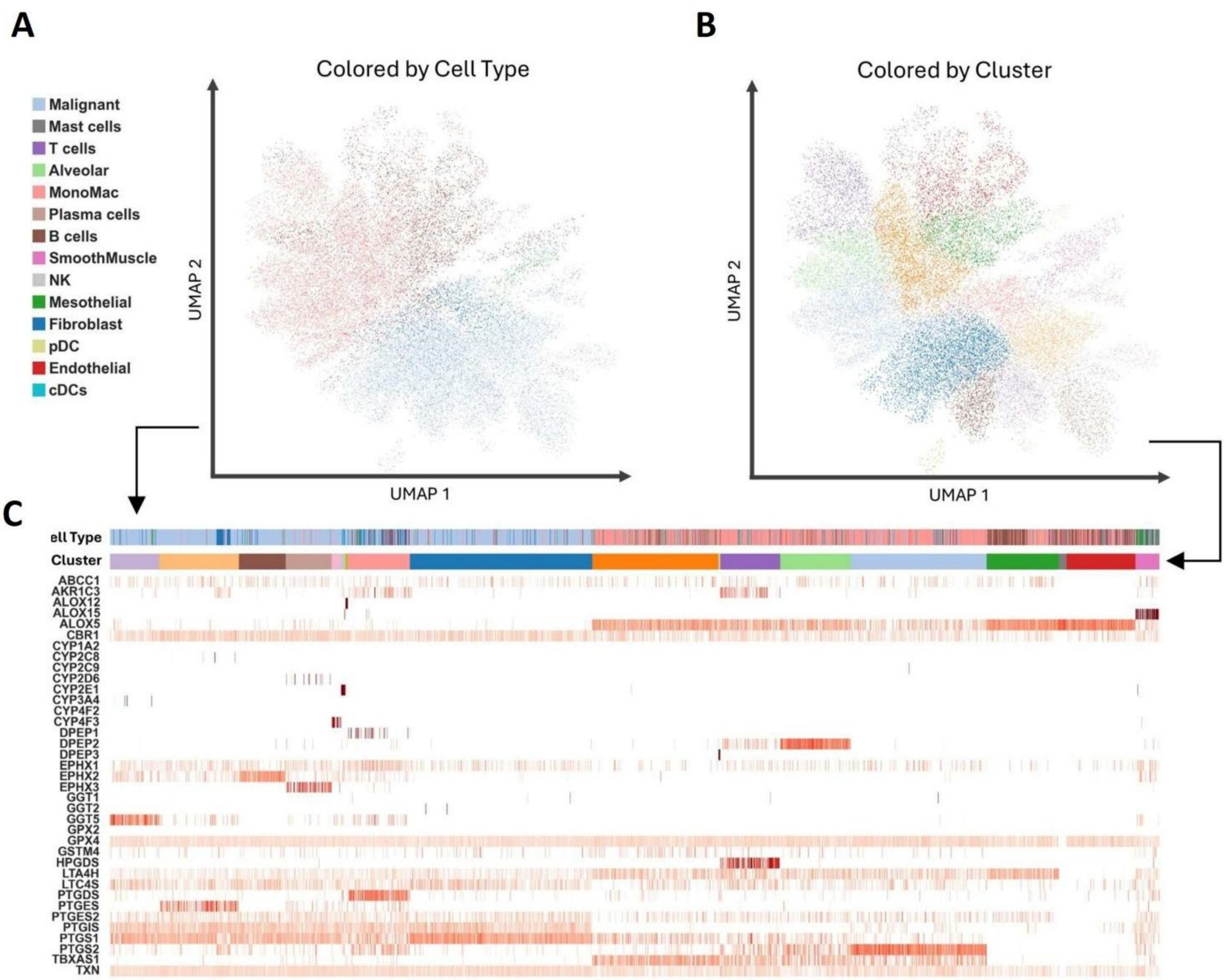
A
B
Colored by Cell Type
Colored by Cluster
Malignant
Mast cells
T cells
Alveolar
MonoMac
Plasma cells
B cells
SmoothMuscle
NK
Mesothelial
Fibroblast
pDC
Endothelial
cDCs
UMAP 2
UMAP 1
C
ell Type
Cluster
ABCC1
AKR1C3
ALOX12
ALOX15
ALOX5
CBR1
CYP1A2
CYP2C8
CYP2C9
CYP2D6
CYP2E1
CYP3A4
CYP4F2
CYP4F3
DPEP1
DPEP2
DPEP3
EPHX1
EPHX2
EPHX3
GGT1
GGT2
GGT5
GPX2
GPX4
GSTM4
HPGDS
LTA4H
LTC4S
PTGDS
PTGES
PTGES2
PTGIS
PTGS1
PTGS2
TBXAS1
TXN

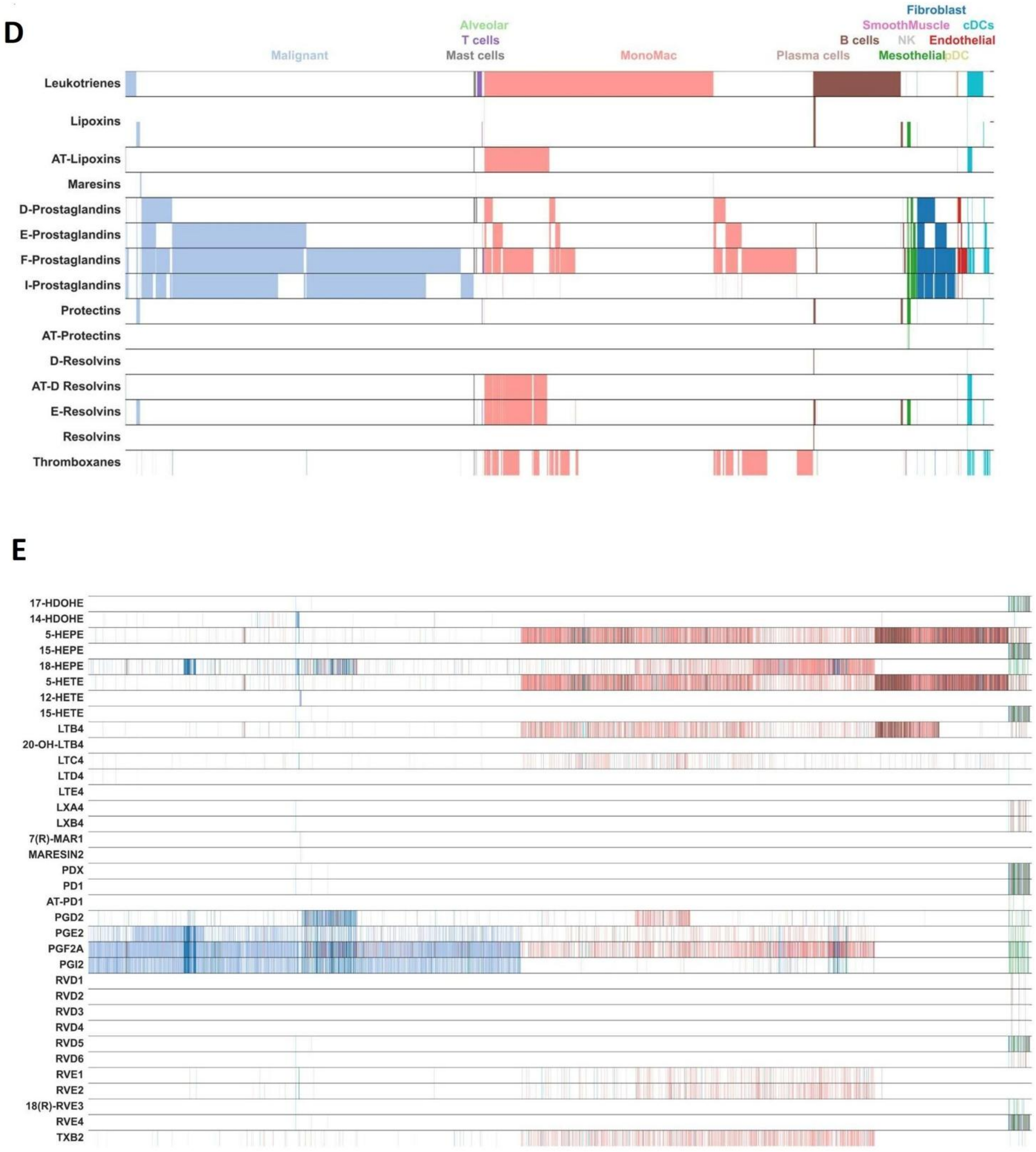

Supplementary Figure 5. Inflammation-resolution biosynthetic pathways of lipid mediators are differentially altered in MPM cell types - predictive model. (A and B) UMAP plots are sorted by cell type and clusters in MPM patients; (C) Heatmaps highlighting SPM enzymes within the cell clusters of MPM patients; (D and E) Heatmaps highlighting individual lipids in cells that express all required enzymes for their synthesis, as measured by targeted lipidomics of MPM patients.

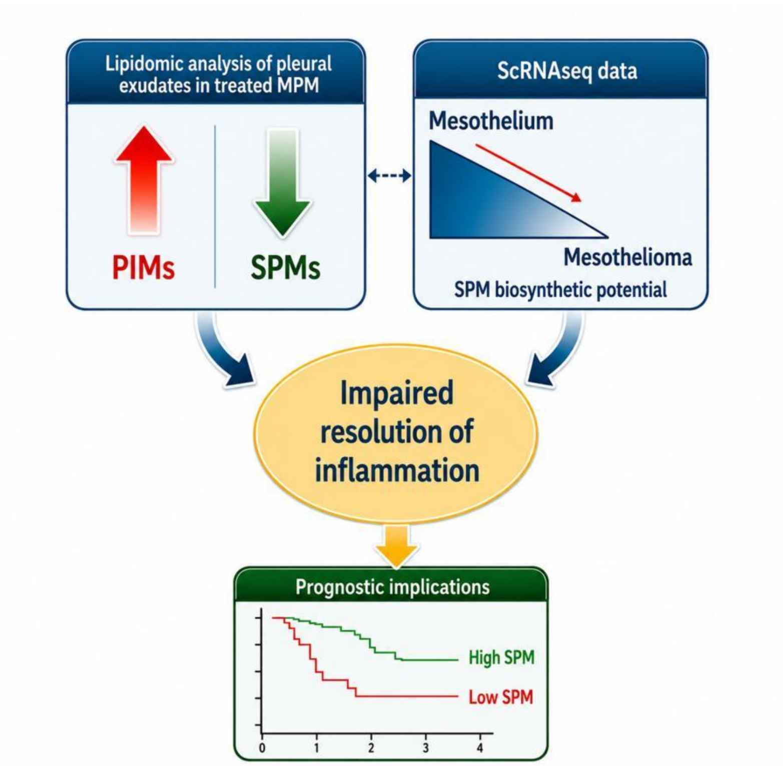

Graphical Abstract. Unresolved inflammation in malignant pleural mesothelioma. The graphical abstract illustrates the imbalance between pro-inflammatory lipid mediators and specialized pro-resolving mediators in malignant pleural mesothelioma. Pleural exudate analysis shows increased prostaglandins in the PIMs compartment and reduced SPMs, including maresins, resolvins, and protectins. Single-cell RNA sequencing data indicate reduced expression of SPM enzymes in mesothelioma cells compared with mesothelial cells. Chemotherapy further decreases SPMs and increases prostaglandin levels, promoting persistent, unresolved inflammation and impaired inflammatory resolution. Low SPM levels are associated with poorer survival outcomes. The AI tool ChatGPT (version 5.5, released 2026-07-08) was used solely for to cosmetically improve the graphical abstract.
